# Identification of *PSMD11* as a novel cuproptosis‐ and immune‐related prognostic biomarker promoting lung adenocarcinoma progression

**DOI:** 10.1002/cam4.7379

**Published:** 2024-06-10

**Authors:** Qiumin Huang, Ran Tian, Jinxi Yu, Wei Du

**Affiliations:** ^1^ Department of Immunology, Biochemistry and Molecular Biology, 2011 Collaborative Innovation Center of Tianjin for Medical Epigenetics, Tianjin Key Laboratory of Medical Epigenetics, Key Laboratory of Immune Microenvironment and Disease of the Ministry of Education Tianjin Medical University Tianjin China; ^2^ Department of Laboratory and Diagnosis Changhai Hospital, Navy Medical University Shanghai China; ^3^ Public Laboratory Tianjin Medical University Cancer Institute and Hospital, National Clinical Research Center for Cancer Tianjin China; ^4^ Tianjin's Clinical Research Center for Cancer Tianjin China; ^5^ Key Laboratory of Breast Cancer Prevention and Therapy Tianjin Medical University, Ministry of Education Tianjin China; ^6^ Key Laboratory of Cancer Immunology and Biotherapy Tianjin China

**Keywords:** cuproptosis, immune cells, lung adenocarcinoma, prognosis, *PSMD11*

## Abstract

**Background:**

Due to the unfavorable prognosis associated with lung adenocarcinoma (LUAD), the development of targeted therapies and immunotherapies is essential. Cuproptosis, an emerging form of regulated cell death, is implicated in mitochondrial metabolism and is induced by copper ions. This study aimed to explore the prognostic value of cuproptosis‐ and immune‐related genes (CIRGs) in LUAD.

**Methods:**

We used The Cancer Genome Atlas database to develop a prognostic prediction model for LUAD patients based on eight CIRGs. Using Cox regression analysis, we determined that the CIRG signature is a reliable, independent prognostic factor. We further identified *PSMD11* as a critical CIRG and performed immunohistochemistry to study the protein expression levels of PSMD11 in LUAD tissues. We also investigated the impact of PSMD11 on the biological behavior of lung cancer cell lines.

**Results:**

We found that patients with low PSMD11 expression levels displayed an improved prognosis compared with those with high PSMD11 expression levels. Overexpression of *PSMD11* enhanced proliferation, migration, invasion, and tumor growth of lung carcinoma cell line A549, while *PSMD11* knockdown diminished proliferation, migration, invasion, and tumor growth of lung carcinoma cell line PC9. Additionally, we discovered that *PSMD11* expression was positively correlated with the infiltration of myeloid‐derived suppressor cells and the increased expression of immunosuppressive molecules.

**Conclusion:**

These findings suggest that *PSMD11* may serve as a valuable prognostic biomarker and therapeutic target for LUAD.

## 1 INTRODUCTION

Lung cancer is one of the most prevalent cancers globally, with a 5‐year relative survival rate of less than 20%.^1^ Lung adenocarcinoma (LUAD) represents a major histological subtype of lung cancer, accounting for approximately 45% of all cases and threatening human health worldwide.^2^ With the development of targeted drugs and immune checkpoint inhibitors, therapeutic options for patients with LUAD have changed dramatically.^3^, ^4^, ^5^ However, the overall survival (OS) rate of patients with LUAD has not shown significant improvement. Therefore, there is an urgent need to explore new sensitive biomarkers to determine the prognosis or develop novel therapies for patients with LUAD.

Regulated cell death (RCD), encompassing apoptosis, autophagy, and necroptosis, plays a crucial role in organismal development, tissue remodeling, cell homeostasis, and disease progression.^6^ Investigating the molecular mechanisms of RCD processes has provided new avenues for cancer therapy.^7^ Cuproptosis is a recently discovered, novel form of the RCD that is closely linked to mitochondrial metabolism and is induced by copper ionophores targeting lipoylated molecules.^8^ Copper ions bind to the lipid‐acylated components of the tricarboxylic acid cycle, leading to abnormal aggregation of lipid‐acylated proteins, a proteotoxic stress response, and finally cell death, in a manner independent of the apoptotic pathway. Tumorigenesis and cancer development are associated with mitochondrial dysfunction,^9^ and numerous studies have demonstrated that suppression of mitochondrial respiration hinders cancer progression.^10^, ^11^ However, the role of cuproptosis in the initiation, development, and prognosis of lung cancer remains unclear.

In this study, we explored the association between cuproptosis‐ and immune‐related genes (CIRGs) and lung cancer prognosis by analyzing a public database. We then established a multi‐gene LUAD prognosis prediction model using LASSO regression and Cox regression analyses. Notably, we identified *PSMD11* as a novel cuproptosis‐ and immune‐related prognostic biomarker. *PSMD11* is a 26S proteasome non‐ATPase regulatory subunit 11, which regulates the breakdown of ubiquitinated proteins and is associated with tumor progression. In urothelial bladder cancer, *PSMD11* is highly expressed in tumor tissues compared with normal tissues.^12^ In pancreatic ductal adenocarcinoma, *PSMD11* expression was associated with a poorer OS.^13^ Here, we elucidated the role of *PSMD11* in LUAD cell proliferation, migration, invasion and tumor growth, and found that it is linked to the recruitment of myeloid‐derived suppressor cells (MDSCs) and the expression of immune checkpoints. Our findings indicated the potential prognostic significance of *PSMD11* in LUAD progression and could provide new insights into LUAD therapy and prognosis.

## 2 MATERIALS AND METHODS

### 2.1 Data acquisition and differential gene analysis

Transcriptome profiling (RNA sequencing [RNA‐seq]) data and corresponding clinical information of 598 samples (59 normal lung tissue and 539 LUAD samples) used for further validation were mined from The Cancer Genome Atlas (TCGA) database (https://portal.gdc.cancer.gov/). The microarray dataset GSE72094 with OS data was obtained from the GEO database (https://www.ncbi.nlm.nih.gov/geo/), comprising 442 GEO‐LUAD cases. The list of identified immune‐related genes was obtained from the Immunology Database and Analysis Portal (ImmPort) database (http://www.immport.org). Cuproptosis‐related genes were identified by summarizing previous literature.^14^ The extracted gene expression profiles from the TCGA and GEO databases were normalized to gene expression data using the limma package in R software. Pearson's correlation analysis was used to identify CIRGs with a *p* value <0.05 and an absolute Pearson correlation coefficient >0.3 or < −0.3.

### 2.2 Construction and validation of the prognostic model

Univariate Cox regression analysis was performed to screen for significant variables and identify their prognostic values using multivariate Cox analysis. To construct the prognostic model, we performed LASSO penalized Cox regression analysis using the glmnet package in R and calculated the risk score. Based on the prognostic signature, each patient's risk score was calculated using the following formula: Risk score = (Coef 1 × expression mRNA 1) + (Coef 2 × expression mRNA 2) + (Coef n × expression mRNA n). Coef is the Cox regression coefficient of the corresponding mRNA. Based on the median value of the risk score, we classified the patients into high‐ and low‐risk groups. An OS curve was plotted using Kaplan–Meier analysis and compared using the log‐rank test. Time‐dependent receiver operating characteristic (ROC) curve analysis was used to determine the prognostic ability and accuracy of the prognostic risk score model.

### 2.3 Functional analyses

Functional enrichment analysis of differentially expressed genes was performed using the clusterProfiler R package to identify Gene Ontology (GO) terms based on biological processes (BP), molecular functions (MF), and cellular components (CC). Kyoto Encyclopedia of Genes and Genomes (KEGG) pathway analysis was conducted using the clusterProfiler package. The cut‐off criterion of the *p* value was set as <0.05 to identify significant pathways.

### 2.4 Construction and validation of a predictive nomogram

By integrating all clinical variables (age, gender, stage, and grade) and risk scores, we constructed a nomogram associated with outcomes for predicting the probability of 1‐, 3‐, and 5‐year OS for patients with LUAD. Calibration curves and a concordance index (C‐index) were generated to evaluate the predictive accuracy of the nomogram.

### 2.5 Immune cell infiltration analysis

Tumor Immune Estimation Resource (TIMER) (https://cistrome.shinyapps.io/timer/) offers a user‐friendly web interface for exploring and visualizing the immune cell composition of tumors. Spearman's correlation analyses were carried out to describe the association between *PSMD11* expression and the proportion of immune cells. We also utilized the TCGA database to investigate the association between *PSMD11* and immune checkpoints. A *p*‐value <0.05 was considered statistically significant in these analyses.

### 2.6 Cell culture

A549 cell line was acquired from the American Type Culture Collection (ATCC, Manassas, VA, USA). PC9 cell line was obtained from the European Collection of Authenticated Cell Cultures (ECACC). These cells were cultured in Roswell Park Memorial Institute 1640 medium (Biological Industries, Beit Haemek, Israel) supplemented with 10% fetal bovine serum (FBS, Biological Industries, Beit Haemek, Israel), and incubated at 37°C in a humidified atmosphere containing 5% CO_2_. The cell lines were authenticated by short tandem repeat analysis in 2023.

### 2.7 Quantitative real‐time transcription (qRT)‐PCR

Following the manufacturer's instructions, total RNA was extracted using Trizol (Invitrogen) reagent from LUAD, adjacent tissues, and cell lines. For reverse transcription, a reverse transcription kit (Invitrogen) was used. Primer sequences are illustrated in Table S1. Real‐time PCR was performed using a SYBR Green PCR assay kit (DBI Bioscience) and an ABI7900 system. All RNA expression levels were normalized to GAPDH.

### 2.8 Immunohistochemical analysis

For PSMD11 immunostaining, tissue paraffin sections of lung specimens were dewaxed in xylene and then rehydrated in a graded concentration of ethanol and distilled water. Endogenous peroxidase activity was blocked with 0.3% H_2_O_2_ at RT for 10 min. Sections were blocked with 10% NGS for 30 min and then incubated overnight at 4°C with an antibody against PSMD11 (Bioworld Technology, 1:100). The next day, following secondary antibody incubation, the peroxidase reaction was performed using diaminobenzidine (DAB). Tissue sections were quantitatively scored according to the percentage of positive cells and staining intensity.

### 2.9 Immunoblots

Cells were lysed with Laemmli buffer on ice. Lysates were collected by scraping the culture dishes, briefly ultrasonicated and boiled for 10 min. Subcutaneous tumor tissues were ground in liquid nitrogen and mixed with 200 μL of cold protein extraction buffer. The tissue extracts were briefly ultrasonicated and boiled for 10 min. Protein samples were separated by 10% SDS‐PAGE, and then electroblotted onto nitrocellulose membranes. Membranes were blocked with 5% nonfat milk for an hour and then incubated with primary antibodies PSMD11 (Bioworld Technology), β‐actin (Millipore, Billerica, MA, USA), or GAPDH (Bioworld Technology) overnight at 4°C. PSMD11 primary antibody was diluted at 1:2000, β‐actin antibody was diluted at 1:5000 and GAPDH antibody was diluted at 1:5000. After being washed three times with TBST, the membranes were incubated with the corresponding secondary antibodies for 2 h at RT. An enhanced chemiluminescence detection substrate (Millipore) was utilized to detect the immunoreactive bands. The densitometric analysis of these bands was conducted using ImageJ software.

### 2.10 Single‐cell RNA‐seq (scRNA‐seq) analysis

We utilized the published data from the NCBI GEO database with the accession code GSE148071.^15^ By analyzing differentially expressed genes in each cluster, we had previously identified 11 major cell types.^16^ The final results were visualized using t‐distributed stochastic neighbor embedding (t‐SNE) for dimensionality reduction.

### 2.11 Plasmids construction and lentiviral transfection

The full‐length *PSMD11* open reading frame (ORF) was amplified using PC9 cDNA. The amplified DNA fragment was ligated into the lentiviral shuttle pCCL.PPT.hPGK.IRES.GFP/pre. DNA oligonucleotides encoding *PSMD11*‐specific shRNA were ligated into the pCCL.PPT.hPGK.GFP.Wpre vector. The primer sequences for the *PSMD11* ORF and the shRNA sequences targeting *PSMD11* are listed in the Table S1.

Lentiviral particles were produced by co‐transfection of HEK293T cells with lentiviral plasmids and the packaging plasmids using polyethylenimine. Cell supernatants were harvested at 24, 48, and 72 h after transfection, then passed through a 0.45‐μm filter (Thermo Scientific) to remove cells and cellular debris. The supernatants were supplemented with polybrene to a final concentration of 10 μg/mL for target cell infection.

### 2.12 Cell proliferation assay

Lung carcinoma cell lines were seeded into 96‐well plates. The cells were then cultured for three different duration periods: 1 day, 2 days, and 3 days. After each respective duration, 10 μL of CCK‐8 reagent was added to each well. The plates were further incubated for 2 h, and the absorbance was measured at 450 nm using a microplate reader.

### 2.13 Wound healing assay

Lung carcinoma cell lines were seeded into 6‐well plates and cultured to achieve 90% confluence on the plates. A 200‐μL pipette tip was used to scratch the cell layer. The cells were then cultured in medium for 24 or 48 h, after which they were observed under a light microscope.

### 2.14 Transwell migration assay

Lung carcinoma cell lines were seeded into 8 μm pore size transwell filters coated with 20% Matrigel. Complete medium was added to the bottom chambers. The cells were cultured for 24 h, after which they were fixed in 4% paraformaldehyde for 15 min, followed by staining with 0.5% crystal violet for another 15 min. The cells were then counted under a light microscope.

### 2.15 Xenograft model

Female BALB/c nude mice (6 weeks old) were provided from Beijing Sibeifu biotechnology Co. Ltd (Beijing, China) and were reared in specific pathogen‐free (SPF) conditions. Tumor cell lines (2 × 10^6^) in 100 μL PBS containing Matrigel (1:1 vol/vol; Corning) were subcutaneously injected into BALB/c nude mice (5 mice per group). 23 days after inoculation, in situ tumors were excised to analyze tumor growth. Animal studies were approved by the Tianjin Medical University Animal Care and Use Committee.

### 2.16 Statistical analysis

All statistical analyses were conducted using the R software (version 4.2.1) and Perl language packages. The Wilcoxon signed‐rank test and Student's *t*‐test were used to compare differences between groups. The correlation between PSMD11 expression levels and survival rates was determined with Kaplan–Meier analysis using Mantel‐Cox log‐rank testing (GraphPad Prism). Statistical significance was defined as *p* < 0.05.

## 3 RESULTS

### 3.1 Establishment and validation of a CIRG prognosis signature

**FIGURE 1:**
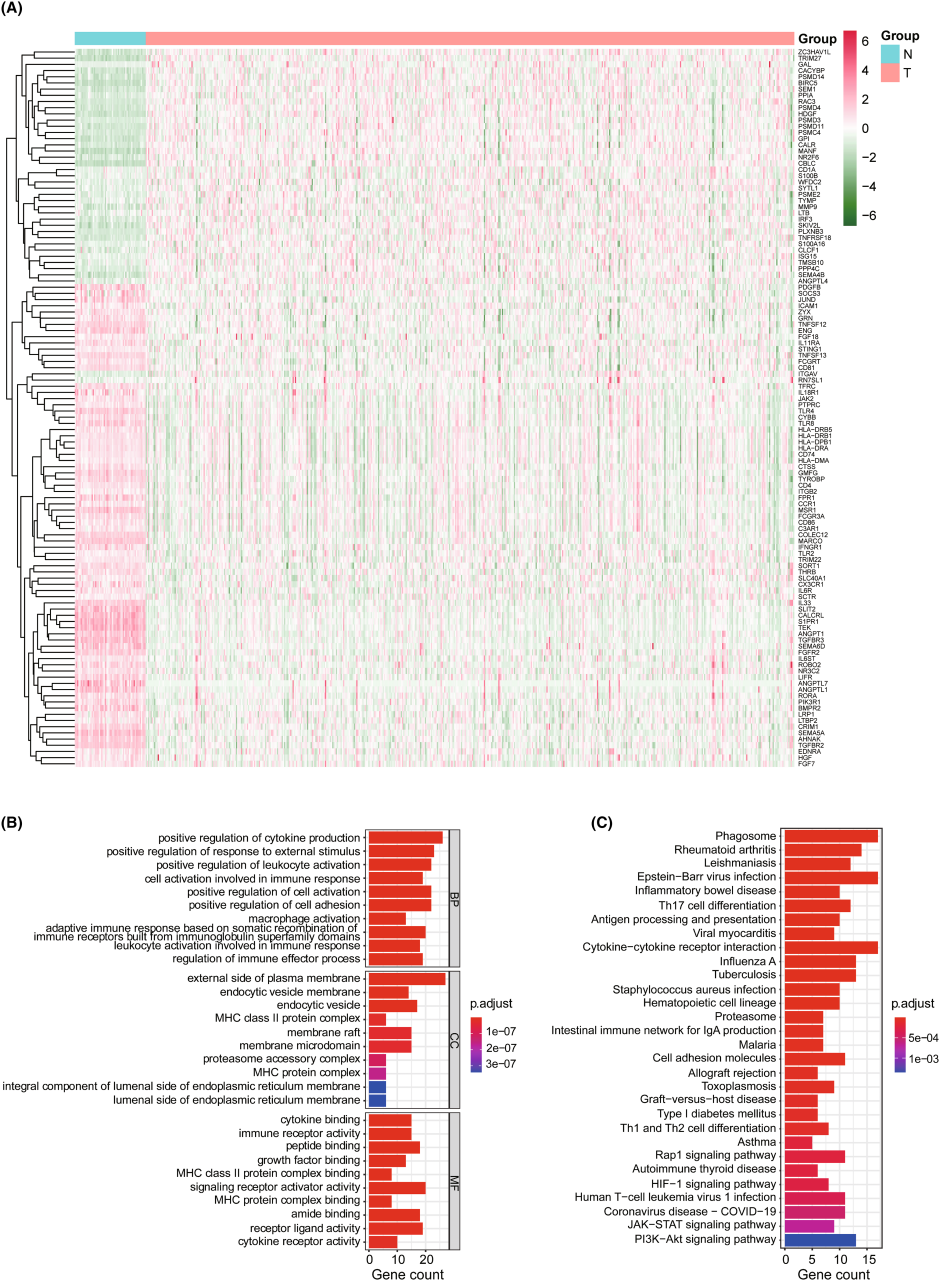
Identification of the differentially expressed CIRGs in Lung adenocarcinoma (LUAD). (A) Heatmap illustration of the expression of differentially expressed genes in LUAD and non‐tumor samples. (B) The top 10 significant terms of Gene Ontology (GO) functional annotation (biological processes [BP]/cellular components [CC]/molecular functions [MF]) of differentially expressed cuproptosis‐ and immune‐related genes. (C) The top 30 significant terms of Kyoto Encyclopedia of Genes and Genomes (KEGG) pathway enrichment analysis.

We analyzed the expression association between 13 cuproptosis‐related genes from previous reports^14^ and 1793 immune‐related genes in 539 LUAD and 59 non‐tumor tissues from TCGA using Pearson's correlation, and identified differential genes using Wilcoxon signed‐rank tests. A total of 116 differentially expressed CIRGs were found using the criteria of |FoldChange| > 1.5 and false discovery rate (FDR) < 0.05, including 40 upregulated genes and 76 downregulated genes in the LUAD dataset compared with the normal lung tissue data (Figure 1A). We performed GO enrichment and KEGG pathway analyses to explore the potential function of differentially expressed CIRGs. We found that the significantly enriched pathways are related to immunity, such as positive regulation of cytokine production, positive regulation of cell adhesion, and regulation of immune effector process (BP); external side of plasma membrane, membrane microdomain, and MHC protein complex (CC); and cytokine binding and immune receptor activity (MF) (Figure 1B). In KEGG enrichment analysis, CIRGs tended to be involved in many pathways, such as antigen processing and presentation, cytokine–cytokine receptor interaction, proteasome, cell adhesion molecules, the Rap1 signaling pathway, the HIF‐1 signaling pathway, and the JAK–STAT signaling pathway (Figure 1C). These results suggest that these 116 CIRGs are important regulators of LUAD progression.

**FIGURE 2:**
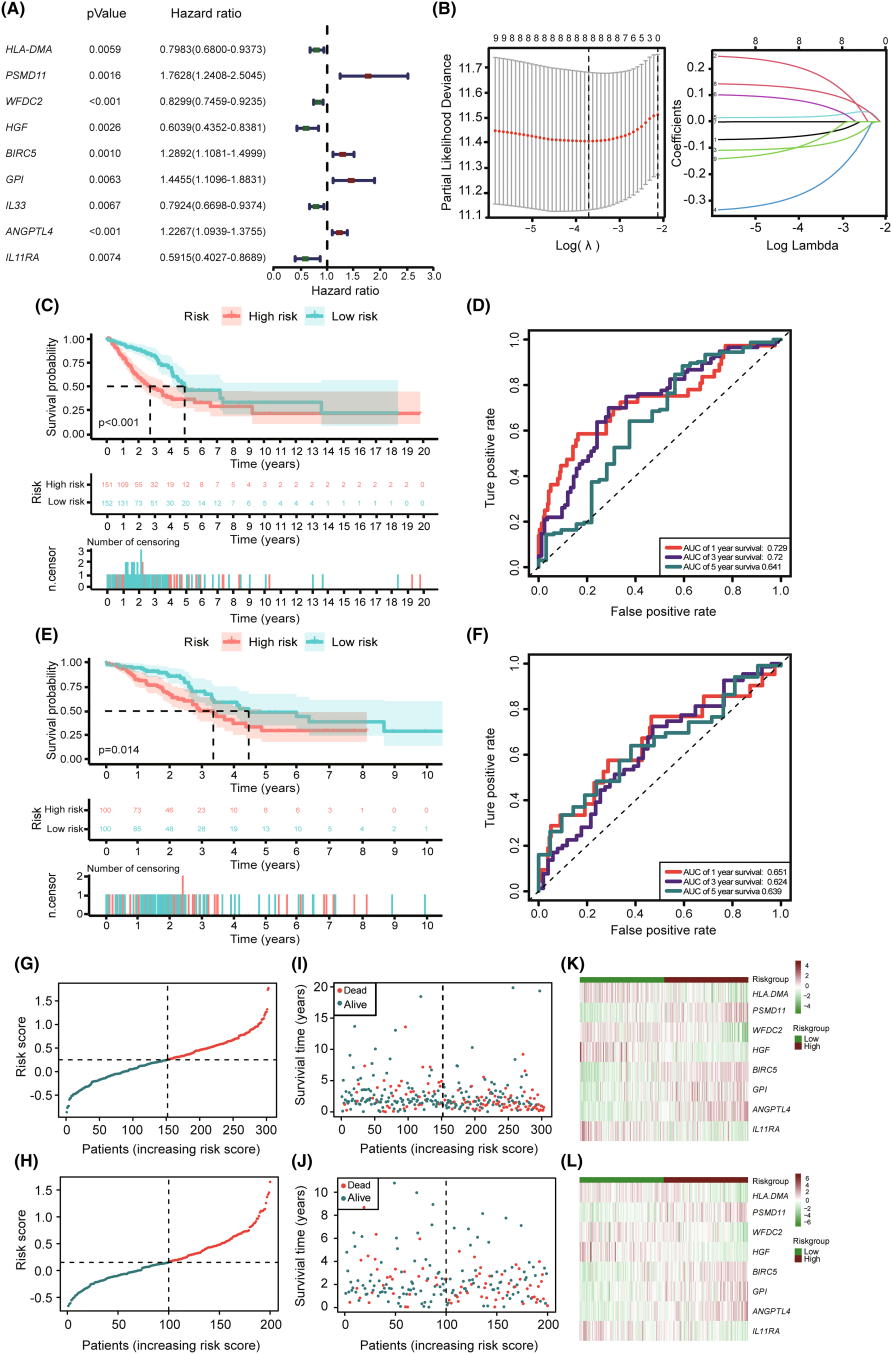
Identification of prognostic CIRGs by univariate Cox regression analysis and construction of the predictive model using a LASSO regression. (A) Univariate Cox regression analysis determined that nine cuproptosis‐ and immune‐related genes had prognostic values. (B) Optimal cuproptosis‐ and immune‐related gene selection in the LASSO regression for constructing a prediction model. Dotted vertical lines were drawn at the optimal values of lambda to define optimal parameter in the LASSO model (left). Eight variables with non‐zero coefficients were selected using the optimal lambda (right). (C) Kaplan–Meier survival analysis of the high‐risk (red) and low‐risk (green) patients with LUAD in the training cohort. (D) The 1‐year (red), 3‐year (purple), and 5‐year (green) receiver operating characteristic (ROC) curves in the training cohort of patients with LUAD. (E) Kaplan–Meier survival analysis of the high‐risk (red) and low‐risk (green) patients with LUAD in the testing cohort. (F) The 1‐year (red), 3‐year (purple), and 5‐year (green) ROC curves in the testing cohort of patients with LUAD. (G) The distribution of risk scores of patients with LUAD (low, green; high, red) based on the risk score model in the training cohort. (H) Distribution of patients' risk scores (low, green; high, red) based on the risk score model in the testing cohort. (I) Scatterplots of the survival status distribution of patients in the training cohort. (J) Scatterplots of the survival status distribution of patients in the testing cohort. (K) Heatmap showing the expression of risk genes in patients with LUAD in the low‐ and high‐risk groups (low, green; high, red) in the training cohort. (L) Heatmap showing the expression of risk genes in patients with LUAD in the low‐ and high‐risk groups (low, green; high, red) in the testing cohort. CIRGs, cuproptosis‐ and immune‐related genes; LUAD, Lung adenocarcinoma; ROC curves, receiver operating characteristic curves.

We randomly divided the entire TCGA‐LUAD cohort into a training cohort (*n* = 303) and a testing cohort (*n* = 200) at a cutoff of 6:4. In our training group, we used univariate Cox regression analysis to explore the prognostic value of the CIRGs. Only nine of these genes had prognostic values (Figure 2A). We then performed a LASSO Cox regression analysis to construct a prognostic gene signature for predicting the OS of patients with LUAD based on the corresponding coefficients. A risk model was constructed using eight CIRGs (Figure 2B). The prognostic risk score was calculated as follows: risk score = (−0.0500 × *HLA‐DMA* expression) + (0.1894 × *PSMD11* expression) + (−0.0922 × *WFDC2* expression) + (−0.2563 × *HGF* expression) + (0.0192 × *BIRC5* expression) + (0.0717 × *GPI* expression) + (0.1191 × *ANGPTL4* expression) + (−0.0775 × *IL11RA* expression).

Based on the median risk score, patients in the training group were equally classified into high‐ and low‐risk groups. Kaplan–Meier analysis clearly demonstrated that the OS of the low‐risk group was significantly longer than that of the high‐risk group (Figure 2C). The ROC curves also showed that area under the curve (AUC) values were 0.729 at 1‐year, 0.72 at 3‐year, and 0.641 at 5‐year (Figure 2D). Furthermore, data from the testing group were used to validate the accuracy of the model. Kaplan–Meier analysis of the testing group also showed a relatively poor prognosis for patients in the high‐risk group (Figure 2E). In 1‐year, 3‐year, and 5‐year ROC curves, AUCs were 0.651, 0.624 and 0.639, respectively (Figure 2F). In addition, the distribution of risk scores and the survival status of patients with LUAD showed that patients with low‐risk scores survived longer in both the training and testing groups (Figure 2G–L).

The accuracy of the model was further validated in an independent GEO dataset (GSE72094). Kaplan–Meier analysis of the GEO dataset also showed a relatively poor prognosis for patients in the high‐risk group (Figure S1A). In 1‐year, 3‐year and 5‐year ROC curves, AUCs were 0.647, 0.655 and 0.75, respectively (Figure S1B). The distribution of risk scores and the survival status of patients with LUAD also showed that patients with low‐risk scores survived longer in the GEO dataset (Figure S1C–E). These results demonstrate the establishment and validation of the prognostic signature for patients with LUAD.

### 3.2 Cuproptosis‐ and immune‐related genes signature is an independent prognostic factor

**FIGURE 3:**
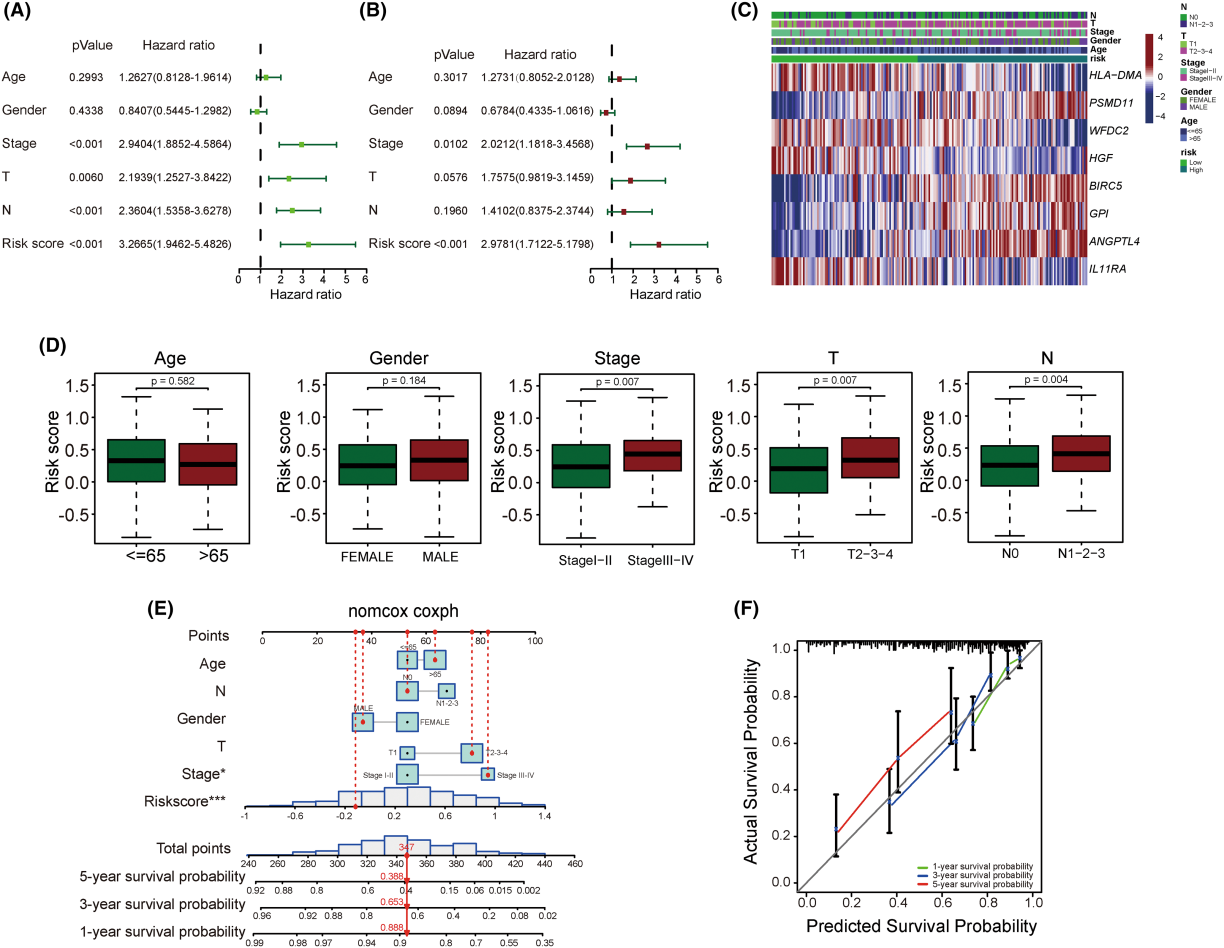
CIRG signature is an independent prognostic factor. (A) Forest plots of univariate Cox regression analysis of the risk score for overall survival (OS) associated with clinicopathological factors. (B) Forest plots of multivariate Cox regression analysis of the risk score for OS associated with clinicopathological factors. (C, D) The correlation between prognosis‐associated signature and clinical characteristics: age, gender, tumor stage, T stage, and N stage. (E) Construction of nomograms integrated with age, gender, and pathological stage for predicting one‐, three‐, and five‐year overall survival probability of patients with LUAD. (F) The calibration plot for validation of the prognostic nomogram. CIRG, cuproptosis‐ and immune‐related gene; LUAD, lung adenocarcinoma.

We then investigated whether the CIRG signature is an independent prognostic factor for LUAD. Univariate Cox regression analysis showed that the risk score (*p* < 0.001), tumor stage (*p* < 0.001), T stage (*p* = 0.006), and N stage (*p* < 0.001) were significantly associated with the survival of patients with LUAD (Figure 3A). Multivariate Cox regression analysis revealed that the risk score (*p* < 0.001) and tumor stage (*p* = 0.0102) were also significantly associated with prognosis (Figure 3B). To explore the association between the signature and clinical characteristics, we used the chi‐squared test. As shown in Figure 3C,D, there were significant differences between the high‐ and low‐risk groups in terms of tumor stage (*p* = 0.007), T stage (*p* = 0.007), and N stage (*p* = 0.004). These results demonstrate that the CIRG signature is an independent prognostic factor for patients with LUAD.

To further predict the survival of patients with LUAD, we created a nomogram model by combining the CIRG risk scores with clinicopathological features (Figure 3E). The calibration curves showed an acceptable fit between the predictions according to the nomogram and the 1‐, 3‐, and 5‐year survival data (Figure 3F). The C‐index of the nomogram for predicting LUAD patient survival was 0.749, which confirmed the favorable predictive ability of the nomogram.

### 3.3 PSMD11 is mainly expressed in lung cancer cells and is associated with poor prognosis in LUAD

**FIGURE 4:**
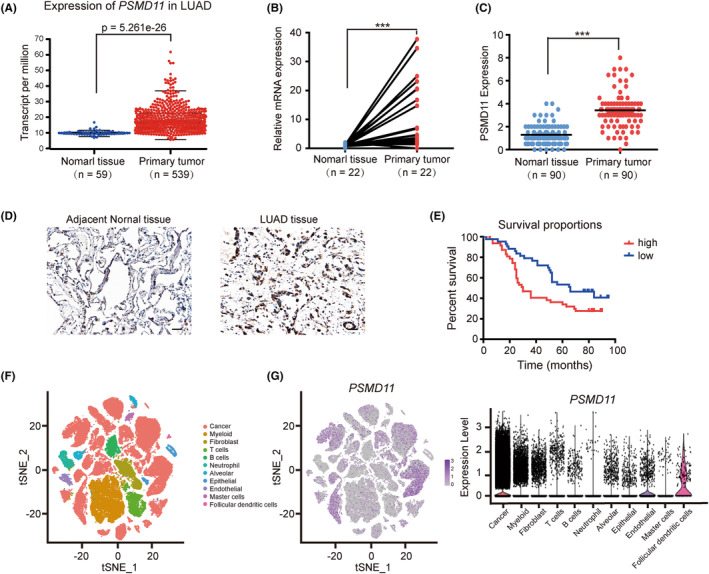
*PSMD11* is mainly expressed in lung cancer cells and is associated with a poor prognosis in lung adenocarcinoma (LUAD). (A) Dot plot displaying *PSMD11* mRNA expression levels in both normal tissues and LUAD tumor tissues, using data from the TCGA database. (B) mRNA expression levels of *PSMD11* were validated through qRT‐PCR in LUAD tumor tissues (*n* = 22) and adjacent tissues (*n* = 22). (C) The protein expression levels of PSMD11 were validated through IHC staining in LUAD tumor tissues (*n* = 90) and adjacent tissues (*n* = 90). (D) Representative images of IHC staining for PSMD11 in LUAD tissues and adjacent normal tissues. Scale bars are 20 μm. (E) Kaplan–Meier survival rates for 90 subjects with LUAD disease with low (staining scores <3.5, *n* = 43, blue line) versus high (staining scores ≥3.5, *n* = 47, red line) PSMD11 expression were compared. Median survivals were 66 months (low PSMD11) versus 30 months (high PSMD11; *p* = 0.0240). (F) t‐SNE analysis shows transcriptionally distinct clusters, with cells colored based on their 11 major cell types. (G) t‐SNE expression plots of *PSMD11* (left) and a violin plot displaying *PSMD11* expression (right). ***p < 0.001.

Utilizing univariate Cox regression analysis, we found that *PSMD11* exhibited a relatively low *p*‐value among the eight CIRGs. *PSMD11* is known to increase 26S proteasome assembly and activity. However, the function of *PSMD11* in LUAD remains unclear. We used TCGA database and qRT‐PCR to evaluate *PSMD11* expression in LUAD. As illustrated in Figure 4A,B, the RNA expression levels of *PSMD11* were significantly higher in LUAD tissues compared with adjacent tissues. We also used IHC analysis to verify the expression of PSMD11 in clinical samples. The results suggested that the expression level of PSMD11 in cancer tissues was significantly higher than that in adjacent normal tissues (Figure 4C,D). Kaplan–Meier survival analysis showed that patients with high PSMD11 expression had poorer OS compared with those with low PSMD11 expression (Figure 4E). Furthermore, PSMD11 expression was found to be associated with gender (*p* = 0.0180) and clinical stage (*p* = 0.0376) (Table 1). High PSMD11 expression was positively correlated with higher clinical stage, suggesting that PSMD11 is associated with tumor progression. Then, we analyzed scRNA‐seq data of NSCLC samples (GSE148071) to compare *PSMD11* expression levels between tumor cells and tumor microenvironment cells. ScRNA‐seq data showed that *PSMD11* was mainly expressed in lung cancer cells, and lowly expressed in other cells (Figure 4F,G). These results suggest that *PSMD11* is predominantly expressed in lung cancer cells and its expression is linked to poor prognosis in LUAD.

**TABLE 1:** The correlation between PSMD11 expression with clinicopathological factors.

	Number of Patients	PSMD11 expression		*p* Value
Parameter		Mean	SEM	
Age				
<60	40	3.5375	0.2464	0.5056
≥60	50	3.3200	0.2142	
Gender				
Male	48	3.7708	0.1895	0.0180^a^
Female	42	3.0119	0.2576	
Clinical stage				
I	44	3.1023	0.2447	0.0376^a^
II + III	46	3.7608	0.1958	
T				
T1	51	3.4118	0.2436	0.9725
T2 + T3 + T4	39	3.4231	0.1957	
N				
N0	56	3.2679	0.2124	0.2382
N1 + N2 + N3	34	3.6618	0.2423	

^a^ Statistically significant.

### 3.4 *PSMD11* promotes the proliferation, migration, and invasion of lung cancer cells in vitro, as well as tumor growth in a subcutaneous mouse model

**FIGURE 5:**
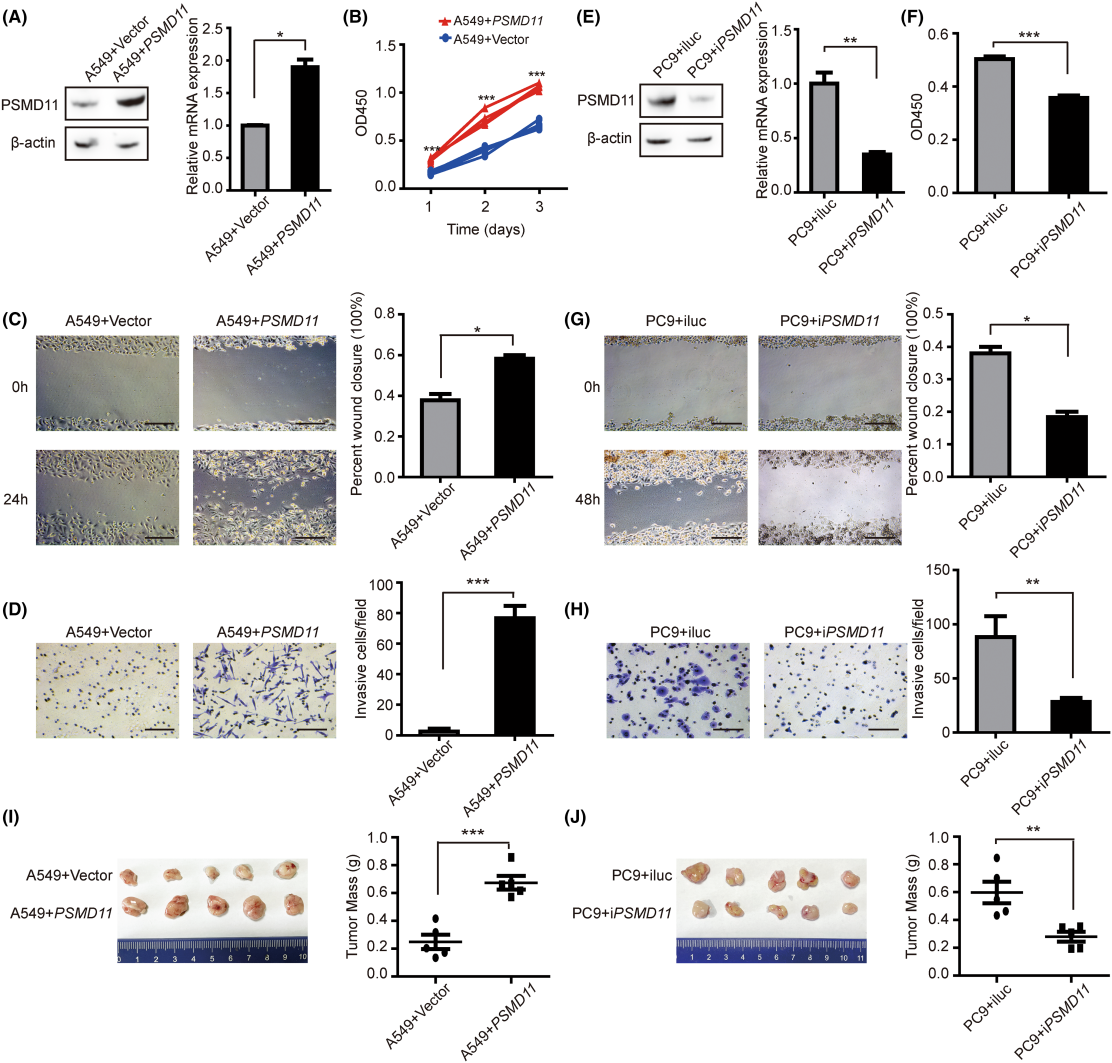
*PSMD11* influences the proliferation, migration, and invasion of lung cancer cells in vitro, as well as tumor growth in a subcutaneous mouse model. (A) The protein and relative mRNA expression of *PSMD11* in A549 cells transfected with *PSMD11‐*expressing or empty vector. (B) CCK‐8 assays demonstrated that overexpression of *PSMD11* promoted A549 cell proliferation. (C) Wound healing assays indicated that *PSMD11* overexpression accelerated A549 cell migration. Scale bars, 100 μm. (D) Transwell invasion assays exhibited enhanced A549 cell invasion upon *PSMD11* overexpression. Scale bars, 100 μm. (E) The protein and relative mRNA expression of *PSMD11* in PC9 cells transfected with vectors expressing shRNA targeting *PSMD11* or control shRNA. (F) CCK‐8 assays demonstrated that *PSMD11* knockdown led to decreased PC9 cell proliferation. (G) Wound healing assays indicated that *PSMD11* knockdown inhibited PC9 cell migration. Scale bars, 100 μm. (H) Transwell invasion assays revealed that *PSMD11* knockdown reduced PC9 cell invasion. Scale bars, 100 μm. (I) A549 cells expressing *PSMD11* or empty vector were subcutaneously injected into 6‐week‐old BALB/c nude mice (*n* = 5, respectively). Left, images of subcutaneous tumors. Right, tumor weight. (J) PC9 cells expressing shRNA against *PSMD11* or control were subcutaneously injected into BALB/c nude mice (*n* = 5, respectively). Left, images of subcutaneous tumors. Right, tumor weight. Mean ± SD. *p < 0.05, **p < 0.01, ***p < 0.001.

To further explore the function of *PSMD11* in LUAD progression, western blotting and RT‐PCR experiments were performed to examine PSMD11 expression in 2 human lung carcinoma cell lines (A549 and PC9, Figure S2). PSMD11 was expressed at a low level in A549, and highly expressed in PC9. Then, we overexpressed *PSMD11* in A549 cells. The protein and mRNA expression levels of *PSMD11* were elevated in the *PSMD11*‐expressing A549 cells than control cells (Figure 5A and Figure S3). Cell proliferation was significantly increased in *PSMD11*‐expressing cells (Figure 5B). Additionally, through wound‐healing and transwell invasion assays, we found that *PSMD11* expression enhances the migration and invasion of A549 (Figure 5C,D).

To confirm the role of *PSMD11* in lung cancer, we knocked down *PSMD11* in PC9 cells (Figure 5E and Figure S3). Results demonstrated that *PSMD11* knockdown significantly reduced PC9 cell proliferation (Figure 5F). Wound‐healing and transwell invasion assays showed that the downregulation of *PSMD11* markedly decreased cell migration and invasion compared with control cells (Figure 5G,H).

To further confirm the function of *PSMD11* in driving lung cancer cells growth, we performed in vivo experiments with subcutaneous tumor formation in 6‐week‐old BALB/c nude mice. 23 days after inoculation, the tumors were excised, and A549 cells with stably expressing *PSMD11* had a superior tumor growth ability compared with vector‐transduced cells (Figure 5I). Consistently, PC9 cells with stably silencing of *PSMD11* had a weaker ability in tumor growth compared with control cells (Figure 5J). Immunoblots have been performed to confirm the expression levels of PSMD11 in subcutaneous tumors (Figure S4). These results indicate that *PSMD11* promotes LUAD progression.

### 3.5 *PSMD11* expression is associated with immune infiltration and immune checkpoint expression in LUAD

**FIGURE 6:**
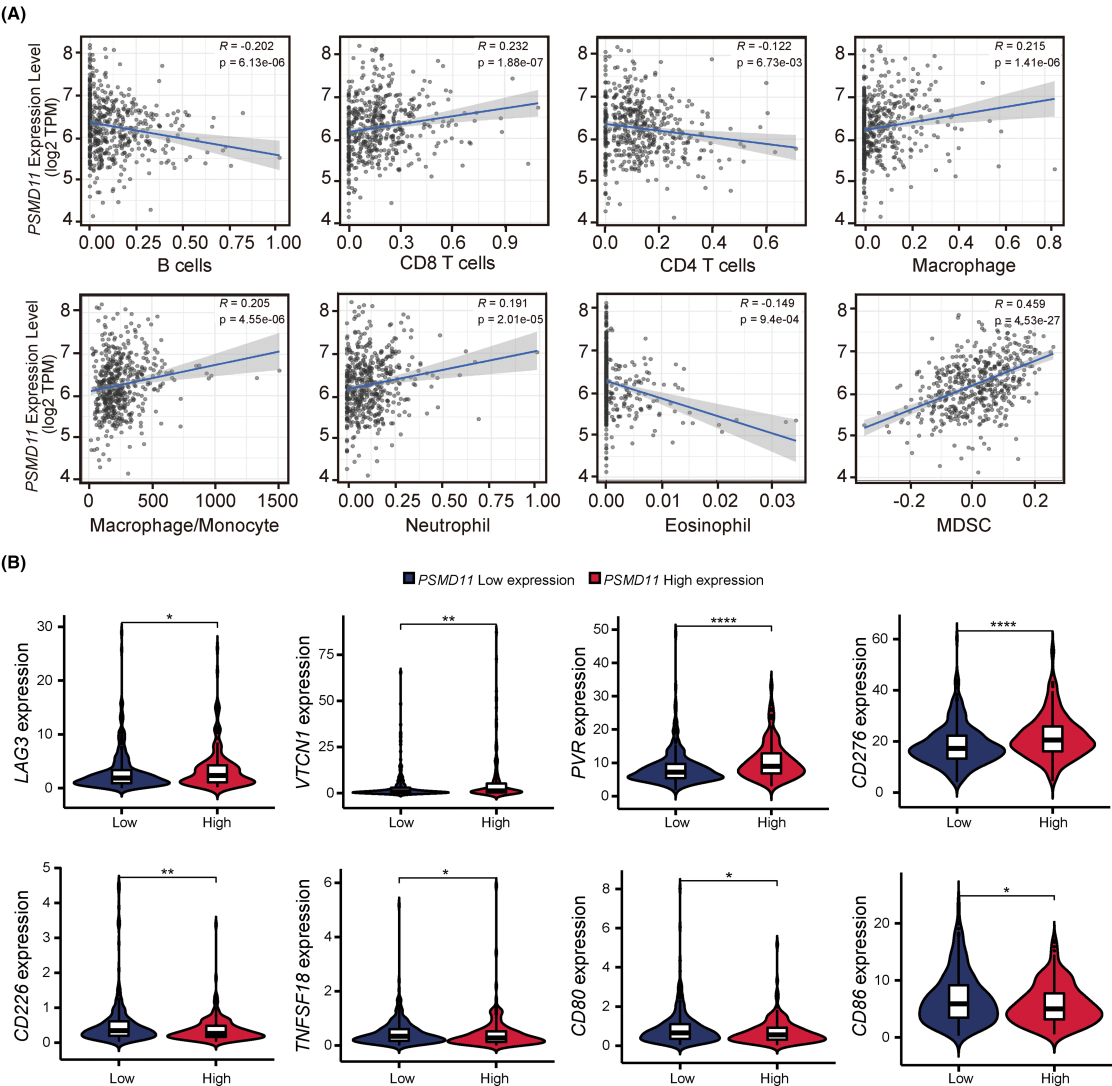
*PSMD11* expression is associated with immune infiltration and immune checkpoint expression in LUAD. (A) The correlation between *PSMD11* expression and the proportions of 8 types of immune cell were shown in LUAD. (B) Box plots display the distribution of immune checkpoint expression in patients with high and low *PSMD11* expression levels. *p < 0.05, **p < 0.01, ***p < 0.001, ****p < 0.0001. LUND, lung adenocarcinoma.

We further explored the correlation between *PSMD11* expression and immune infiltration in LUAD using the TIMER database. The data revealed that *PSMD11* expression had a small negative correlation with B cells, CD4 T cells and eosinophil, and had a small positive association with CD8 T cells, macrophage, macrophage/monocyte and neutrophils (Figure 6A). Furthermore, it had a moderate positive association with MDSCs (Figure 6A). We then investigated the relationship between *PSMD11* expression and immune checkpoints using TCGA database. Our findings showed that immunosuppressive molecules (LAG3, VTCN1, PVR, and CD276) were significantly higher in patients with high *PSMD11* levels, while immune activating molecules (CD226, TNFSF18, CD80 and CD86) were notably lower in patients with high *PSMD11* levels (Figure 6B). These results indicate that *PSMD11* expression plays a crucial role in maintaining an immunosuppressive microenvironment in LUAD.

## 4 DISCUSSION

Cell death can be classified as an accidental cell death (ACD) and RCD by the Nomenclature Committee on Cell Death.^17^ ACD refers to the instantaneous and catastrophic death of cells exposed to natural factors, including physical, chemical, and mechanical stressors.^18^ RCD, also called programmed cell death, relies on a specialized molecular mechanism, suggesting that it can be regulated by pharmacological or genetic interventions.^18^, ^19^ Based on different morphotypes, RCD can be divided into three categories: autophagy, apoptosis, and other types of RCD (e.g., necroptosis, pyroptosis, ferroptosis, and cuproptosis).^20^ Caspase‐dependent apoptosis has long been targeted by anticancer drugs.^21^, ^22^, ^23^ However, some cancer cells develop resistance to drugs and escape apoptosis.^24^ Therefore, exploring new targets from other forms of RCD has become a novel approach to eliminate cancer cells and reduce drug resistance.

A recent study by Tsvetkov et al.^8^ revealed that cuproptosis is a copper‐dependent form of RCD that relies on mitochondrial respiration. Thirteen cuproptosis‐related genes were identified: *FDX1*, *LIPT1*, *LIAS*, *DLD*, *DBT*, *GCSH*, *DLST*, *DLAT*, *PDHA1*, *PDHB*, *ATP7A*, *ATP7B*, and *SLC31A1*. Numerous studies have demonstrated molecular interactions between RCD‐ and immunity‐related genes in tumor tissues. Pyroptosis, a gasdermin‐mediated RCD, exhibits a double‐edged sword effect on tumor progression.^25^ Pyroptosis‐induced cytokines facilitate evasion of immune surveillance, while they also recruit immune cells to help tumor immunotherapy. Wen et al.^26^ found that HMGB1 was released during ferroptotic cell death, inducing the secretion of tumor necrosis factor α (TNFα) from macrophages. Thus, it is crucial to further investigate the interaction network between cuproptosis and immune molecules in tumors. In this study, we assessed the association between cuproptosis and immune‐related genes in LUAD, and identified 116 differentially expressed CIRGs. GO and KEGG enrichment analyses of these differentially expressed CIRGs were performed to explore the molecular mechanisms. GO enrichment analysis revealed that the CIRGs were mainly involved in cytokine production, regulation of cell adhesion, membrane microdomains, and MHC protein complexes. KEGG analysis showed that CIRGs were mainly enriched in cytokine‐cytokine receptor interaction, cell adhesion molecules, Rap1 signaling pathway, HIF‐1 signaling pathway, and JAK–STAT signaling pathway. These results suggest that cuproptosis may be involved in the crosstalk between tumor cells and immune cells.

We then randomly divided the entire TCGA‐LUAD dataset into training and testing sets. Using LASSO regression and Cox regression analyses, we developed a risk model based on eight prognostic CIRGs (*HLA‐DMA*, *PSMD11*, *WFDC2*, *HGF*, *BIRC5*, *GPI*, *ANGPTL4*, and *IL11RA*). Survival and ROC analyses demonstrated that the risk model has good predictive ability. Univariate and multivariate Cox analyses showed that the risk model based on the eight CIRGs is an independent prognostic factor for patients with LUAD.

WFDC2 (also known as HE4) is mainly expressed in pulmonary epithelial cells and may be involved in innate immunity of the respiratory tract.^27^ ANGPTL4 is highly expressed in many cancers and participates in lipid and glucose metabolism.^28^ In lung cancer, ANGPTL4 promotes tumor progression by regulating glutamine and fatty acid metabolism.^29^ BIRC5 (also known as survivin) is usually expressed in embryonic tissues^30^ and plays a role in cell proliferation, angiogenesis, and the negative regulation of apoptosis and autophagy.^31^ Yang et al.^32^ also found that BIRC5 promoted the proliferation of lung cancer. HGF (Hepatocyte Growth Factor) is secreted by mesenchymal cells and could bind to hepatocyte growth factor receptor to regulate cell proliferation and survival.^33^ Although controversy surrounds the prognostic biomarker role of HGF in different types of cancer,^34^ some studies have shown that high expression of HGF predicts improved survival of patients with LUAD.^35^, ^36^, ^37^ HLA‐DMA is a nonclassical MHC II molecule associated with antigen presentation in immune cells. HLA‐DMA catalyzes peptide exchange on classical MHC II molecules and protects empty MHC class II proteins from functional inactivation and disintegration.^38^ In breast cancer, tumor cell expression of HLA‐DMA is positively associated with Th1 infiltration and predicts improved patient survival.^39^ GPI (glucose‐6‐phosphate isomerase) is a moonlighting protein that functions as a cytosolic enzyme involved in glycolysis^40^ and as a cytokine that binds to its receptor.^41^ GPI transamidase subunits contribute to tumor invasion through paxillin phosphorylation.^42^ GPI is highly expressed in several cancers, resulting in poor prognoses.^43^, ^44^, ^45^, ^46^ IL11RA is mainly expressed in stromal and parenchymal cells. IL‐11 stimulates the transformation of lung fibroblasts to myofibroblasts.^47^, ^48^ In this study, utilizing univariate Cox regression analysis, we discovered that the *p*‐value of *PSMD11* was relatively low among the eight prognostic CIRGs, and the role of *PSMD11* in LUAD remains to be elucidated. The expression levels of *PSMD11* were validated by qRT‐PCR and IHC to be significantly different between tumor and normal tissues. We observed a significant association between PSMD11 expression and gender. Tumors from male patients exhibited increased PSMD11 expression. Using the GEPIA database, we analyzed *PSMD11* expression in lung cancer and found higher levels of *PSMD11* expression in lung squamous carcinomas (LUSC) compared with LUAD (Figure S5). Typically, LUSC is more prevalent among male smokers, which could explain the gender association with PSMD11 expression. Single‐cell RNA‐seq data analysis revealed that *PSMD11* was predominantly expressed in lung cancer cells, while exhibiting low expression levels in tumor microenvironment cells. We further investigated the biological function of *PSMD11* in LUAD. The results demonstrated that high expression of *PSMD11* in lung cancer cells facilitated cell proliferation, migration, invasion, and tumor growth in xenograft models. Additionally, we discovered that *PSMD11* expression was linked to the recruitment of MDSCs and the expression of immunosuppressive molecules, suggesting that *PSMD11* expression contributed to maintaining an immunosuppressive microenvironment in LUAD.

The limitation of our study is the absence of exploration into the upstream and downstream mechanisms of *PSMD11*. A previous report suggests that miR‐451 suppresses the proliferation of glomerular mesangial cells by down‐regulating *PSMD11*.^49^ However, the regulatory mechanism of *PSMD11* in cancer remains poorly documented. Further investigation into the regulatory mechanism of *PSMD11* in cancer is warranted.

## 5 CONCLUSION

In summary, we identified a novel prognostic LUAD signature based on eight CIRGs. *PSMD11* is a crucial member of the eight prognostic CIRGs, and we then investigated the function of *PSMD11* in LUAD. *PSMD11* not only promoted tumor cell proliferation, migration and invasion but also influenced the immune infiltration within tumor tissues. These findings suggest that *PSMD11* could serve as a potential biomarker for the prognosis and treatment of patients with LUAD.

## AUTHOR CONTRIBUTIONS

**Qiumin Huang:** Data curation (equal); formal analysis (equal); investigation (equal); methodology (equal); visualization (equal); writing – review and editing (equal). **Ran Tian:** Data curation (equal); formal analysis (equal); funding acquisition (supporting); methodology (equal); resources (supporting); writing – review and editing (equal). **Jinxi Yu:** Data curation (equal); investigation (equal); methodology (equal); writing – review and editing (equal). **Wei Du:** Conceptualization (lead); data curation (equal); funding acquisition (supporting); investigation (lead); methodology (lead); supervision (supporting); validation (lead); writing – original draft (supporting); writing – review and editing (supporting).

## FUNDING INFORMATION

This work was supported by the National Natural Science Foundation of China (grant number 81872319 to W.D., 81903092 to R.T.), and the Tianjin Health Research Project (KJ20174 to R.T.), and Tianjin Key Medical Discipline (Specialty) Construction Project (TJYXZDXK‐010A to R.T.).

## CONFLICT OF INTEREST STATEMENT

The authors have no conflict of interest.

## ETHICS STATEMENT

The studies involving all human lung cancer tissues were approved by the ethics committee of Tianjin Medical University Cancer Institute and Hospital (bc2020178).

## Supporting information


Figure S1.

Table S1.

## Data Availability

All data used in this study were acquired from The Cancer Genome Atlas (TCGA) database (https://portal.gdc.cancer.gov/) and NCBI GEO database (GSE72094 and GSE148071).
