# Supplementary material for: Identification of PSMD11 as a novel cuproptosis‐ and immune‐related prognostic biomarker promoting lung adenocarcinoma progression
Source: Cancer Med. 2024 Jun 10;13(11):e7379. doi: 10.1002/cam4.7379 (PMC11165170; doi:10.1002/cam4.7379)
Supplement: Supplementary file 1 — Figure S1. [file CAM4-13-e7379-s001.docx]

**Identification of *PSMD11* as a novel cuproptosis- and immune-related prognostic biomarker promoting lung adenocarcinoma progression**

Qiumin Huang^1,2§^, Ran Tian^3,4,5,6§^, Jinxi Yu^1^, Wei Du^1*^


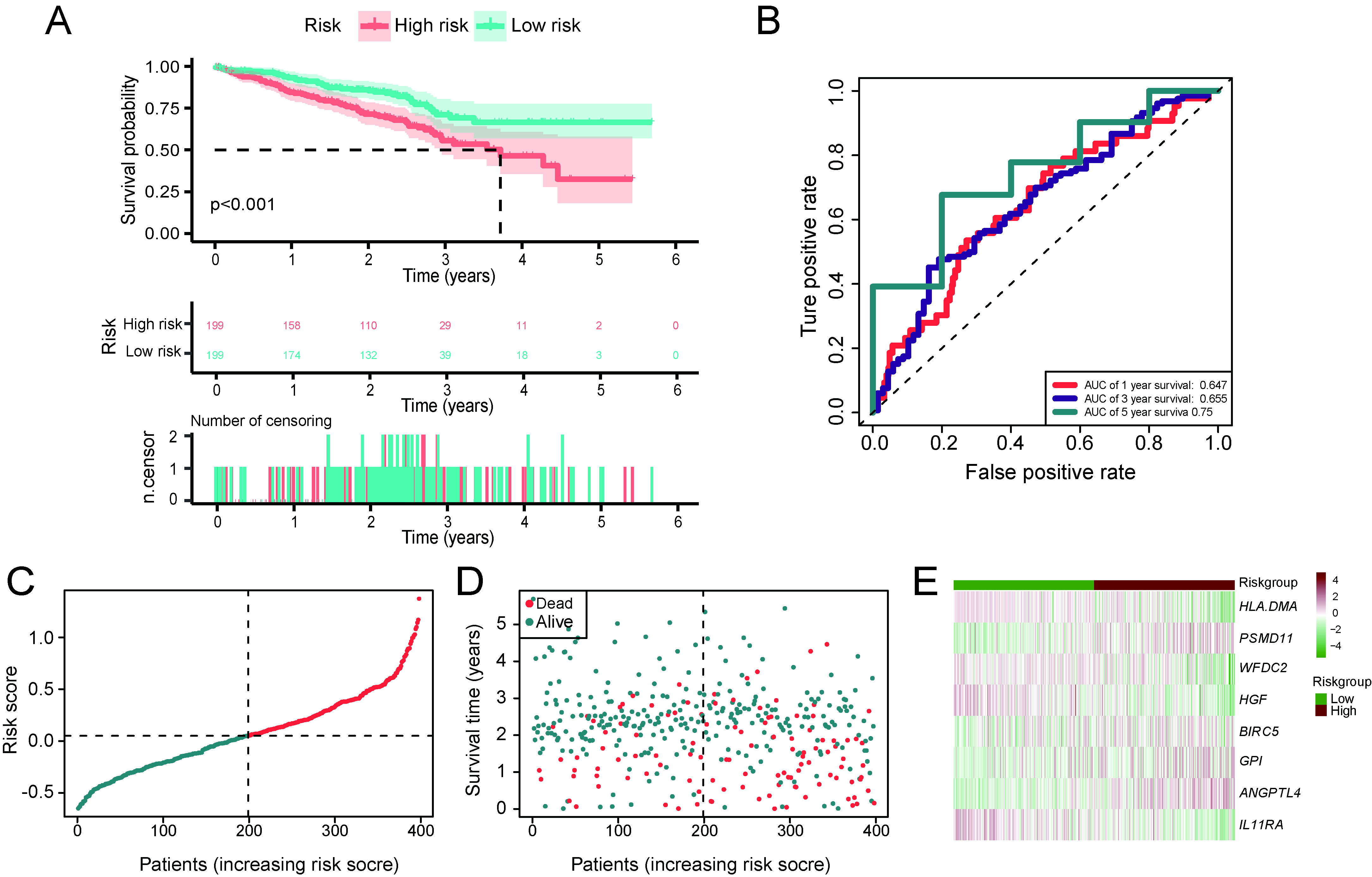


**Figure S1** Validation of CIRGs signature. (A) Kaplan–Meier survival analysis of the high-risk (red) and low-risk (green) patients with LUAD in GSE72094. (B) The 1-year (red), 3-year (purple), and 5-year (green) ROC curves of patients with LUAD in GSE72094. (C) The distribution of risk scores of patients with lung adenocarcinoma (LUAD) (low, green; high, red) based on the risk score model in GSE72094. (D) Scatterplots of the survival status distribution of patients in GSE72094. (E) Heatmap showing the expression of risk genes in patients with LUAD in the low- and high-risk groups (low, green; high, red) in GSE72094.


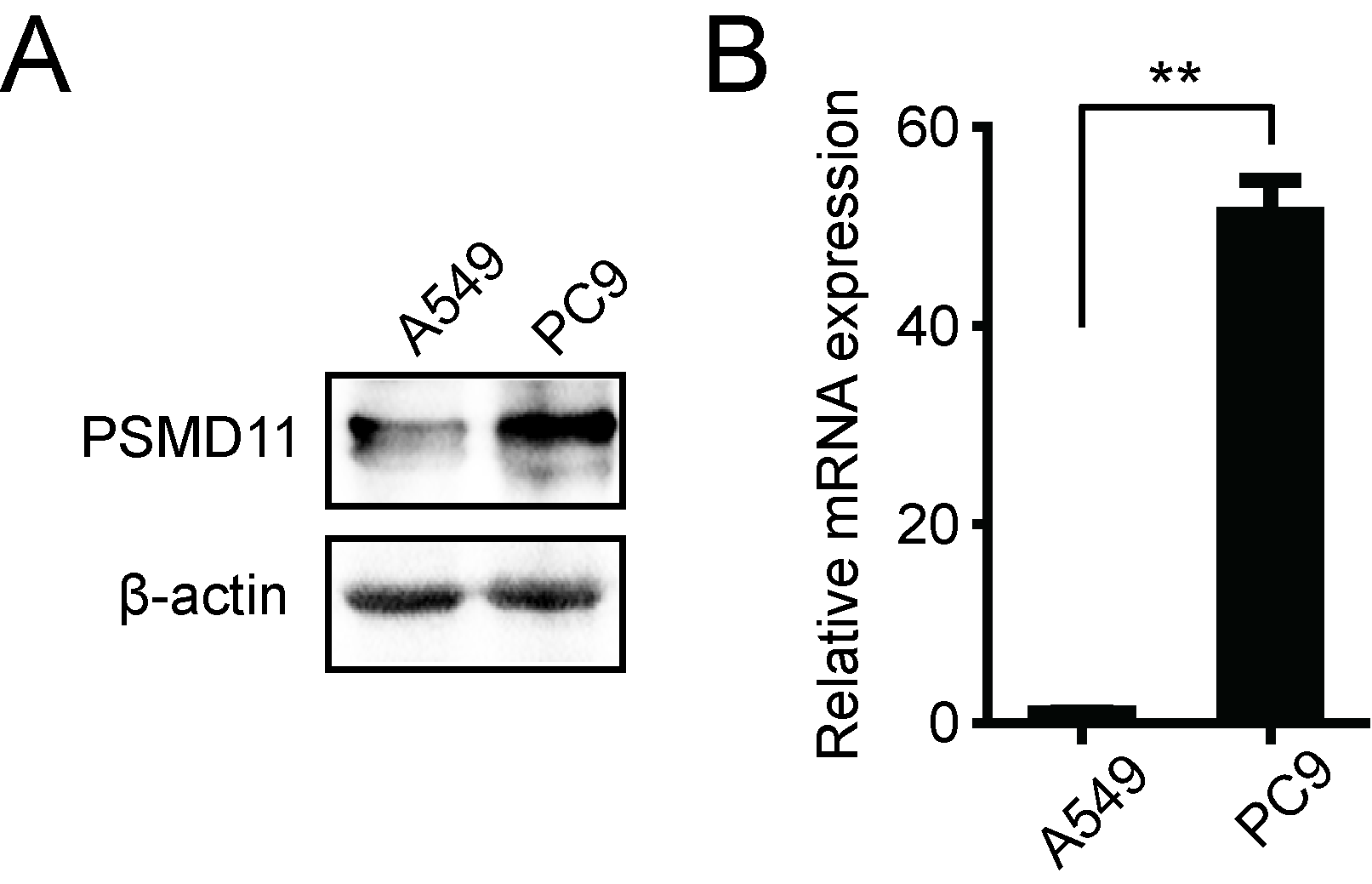


**Figure S2** The protein and relative mRNA expression of *PSMD11* in A549 and PC9 cells. (A) The protein expression of PSMD11 in A549 and PC9 cells. (B) The relative mRNA expression of *PSMD11* in A549 and PC9 cells. **, *p* < 0.01.


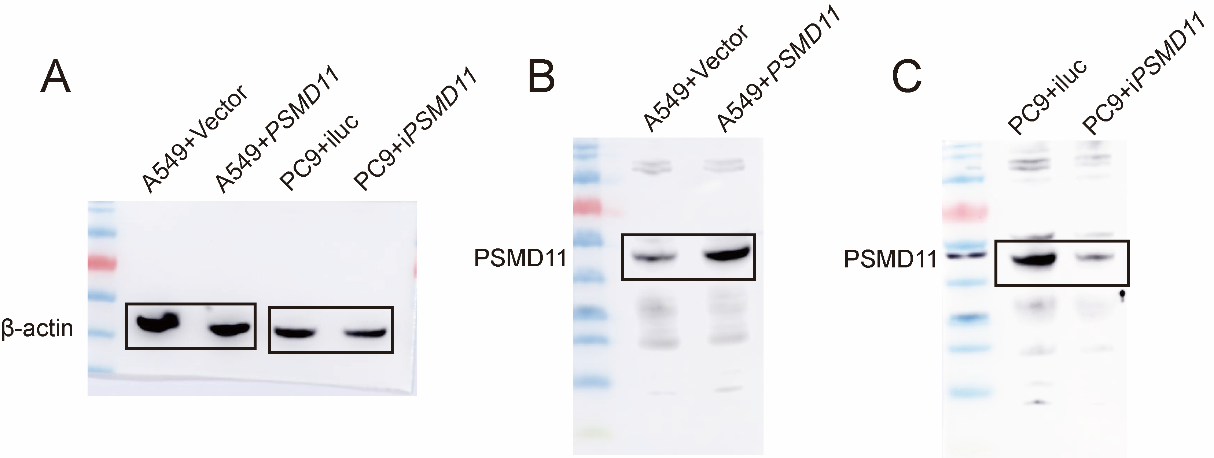


**Figure S3** Immunoblots show expression of β-actin (A) and PSMD11 (B and C).


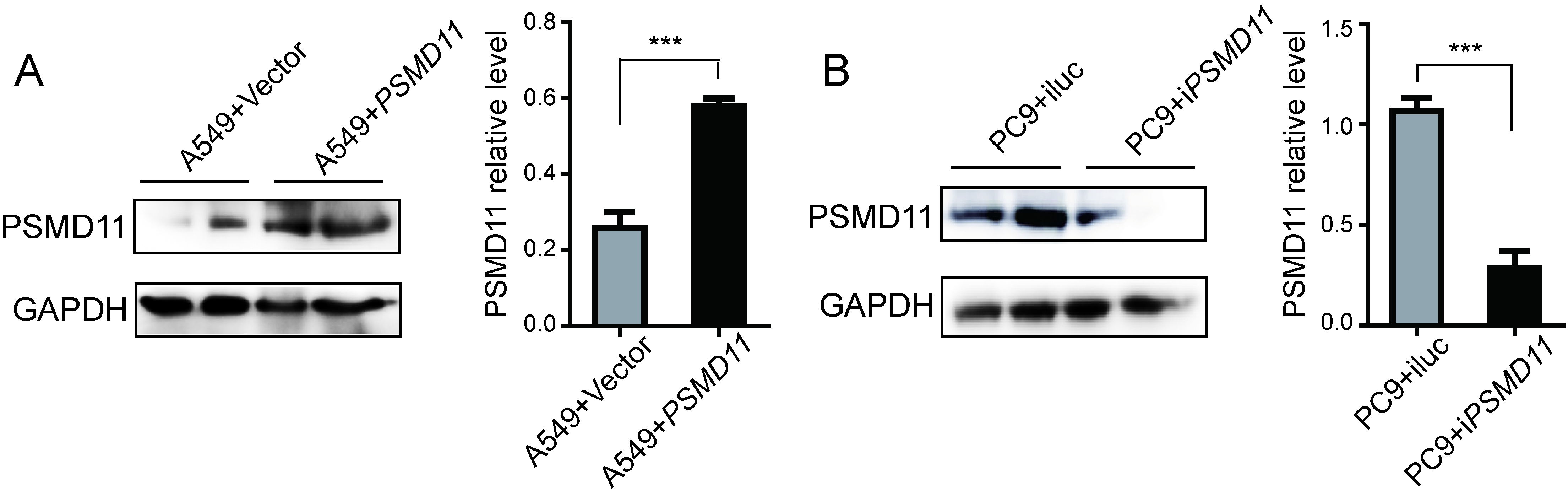


**Figure S4** Immunoblots show PSMD11 protein expression in subcutaneous tumors. (A) The protein expression of PSMD11 in subcutaneous tumors of *PSMD11*-expressing A549 cells and vector-transduced cells. Right, PSMD11 relative protein level. (B) The protein expression of PSMD11 in subcutaneous tumors of *PSMD11*-shRNA knockdown PC9 cells and control cells. Right, PSMD11 relative protein level. The protein expression levels of PSMD11 and GAPDH were determined by densitometric analysis of the protein bands on western blots. Mean ± SD. ***, *p* < 0.001.


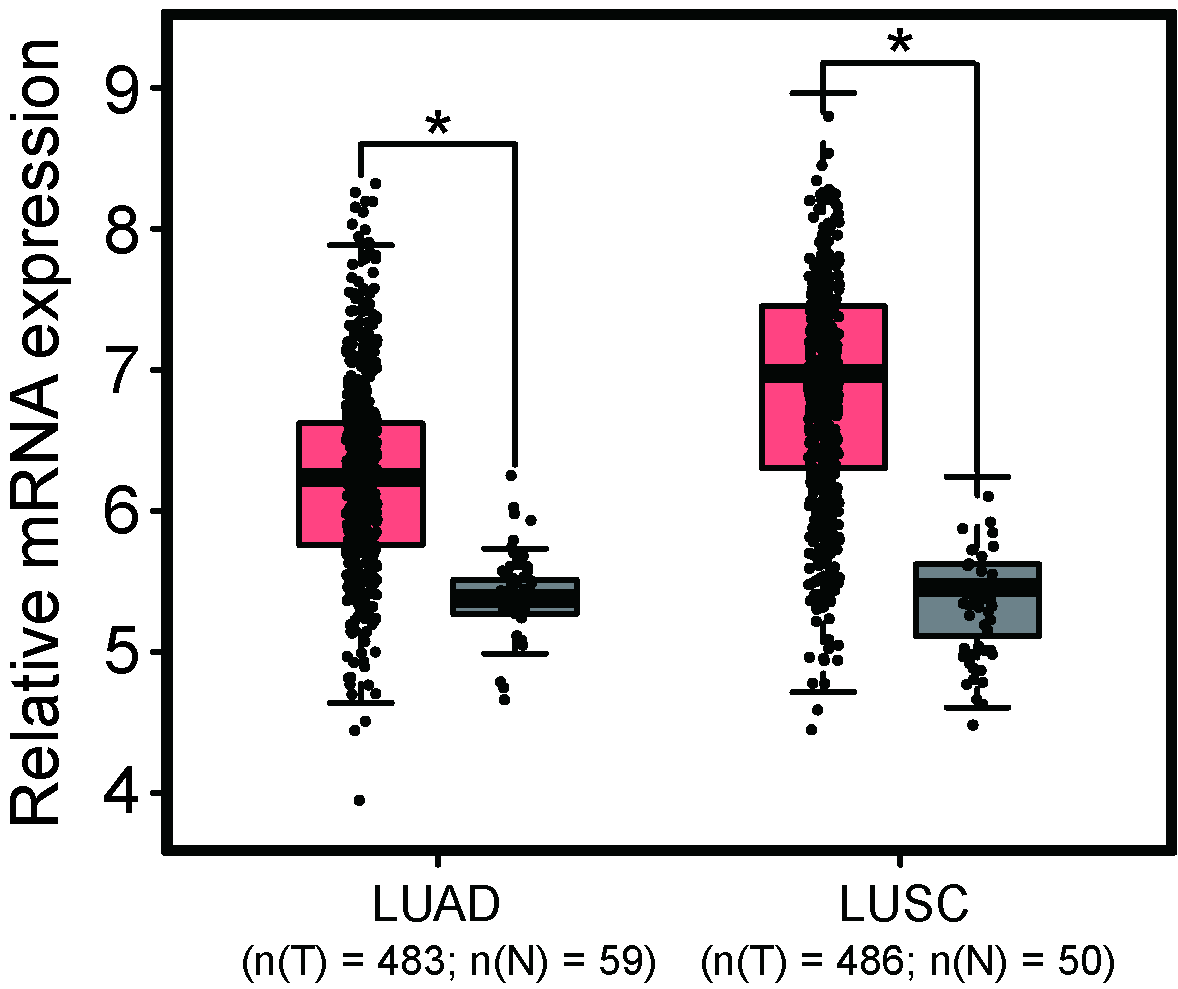


**Figure S5** GEPIA database shows the relative mRNA expression levels of *PSMD11* in LUAD and lung squamous carcinomas (LUSC). *, *p* < 0.05.
