# Supplementary material for: Identification of PSMD11 as a novel cuproptosis‐ and immune‐related prognostic biomarker promoting lung adenocarcinoma progression
Source: Cancer Med. 2024 Jun 10;13(11):e7379. doi: 10.1002/cam4.7379 (PMC11165170; doi:10.1002/cam4.7379)
Supplement: Supplementary file 2 — Table S1. [file CAM4-13-e7379-s002.docx]

**Table S1: Primer sequences**

| Primers used for Quantitative Real-Time Transcription (qRT)-PCR |
| --- |
| h*PSMD11*-F……5’ GCA GCA GAA GAG AAG GAC TGG A 3’ |
| h*PSMD11*-R……5’GCT CAC CAA AGC CTG GAC ATC T 3’ |
| h*GAPDH*-F……5’ GGT GGT CTC CTC TGA CTT CAA CA 3’ |
| h*GAPDH*-R……5’ GTT GCT GTA GCC AAA TTC GTT GT 3’ |
| Primers used for amplifying *PSMD11* ORF |
| *PSMD11*-ORF-F……5’ ACC GAC CTC TCT CCC CAG GGG CCA CCA TGG CGG CGG CGG CGG TGG 3’ |
| *PSMD11*-ORF-R……5’ ACT CTA GAC TCG ACT CTA GAC TAT GTC AGT TTC TTG GCT 3’ |
| shRNA sequences for *PSMD11* |
| *shPSMD11-*F……5’ GAT CCC CCC GAC GTG GAA AGG AAA TTA TTT TCA AGA GAA ATA ATT TCC TTT CCA CGT CGG TTT TTA 3’  *shPSMD11-*R……5’ AGC TTA AAA ACC GAC GTG GAA AGG AAA TTA TTT CTC TTG AAA ATA ATT TCC TTT CCA CGT CGG GGG 3’ |
